# An Anatomical Study on the Measurement of Stature From Ulnar Length in the Adult Ethnic Khasi Tribal Population of the North Eastern Region of India

**DOI:** 10.7759/cureus.22088

**Published:** 2022-02-10

**Authors:** Amitav Sarma, Gautam C Das, Bhupen Barman, Amar J Patowary, Amarantha Donna Ropmay, Polina Boruah, Arup Baruah, Bishwajeet Saikia, Lomtu Ronrang, Ellora Barman

**Affiliations:** 1 Anatomy, North Eastern Indira Gandhi Regional Institute of Health and Medical Sciences, Shillong, IND; 2 Anatomy, Silchar Medical College and Hospital, Shillong, IND; 3 Internal Medicine, North Eastern Indira Gandhi Regional Institute of Health and Medical Sciences, Shillong, IND; 4 Forensic Medicine, North Eastern Indira Gandhi Regional Institute of Health and Medical Sciences, Shillong, IND; 5 Biochemistry, North Eastern Indira Gandhi Regional Institute of Health and Medical Sciences, Shillong, IND; 6 General Surgery, North Eastern Indira Gandhi Regional Institute of Health and Medical Sciences, Shillong, IND; 7 Dentistry, North Eastern Indira Gandhi Regional Institute of Health and Medical Sciences, Shillong, IND; 8 Library Science, North Eastern Institute of Ayurveda and Homeopathy, Shillong, IND

**Keywords:** northeast india, anthropometry, khasi, stature, ulna

## Abstract

Introduction

The stature of an individual is an important parameter for establishing identification. The height of an individual can be indirectly estimated from different parts of the skeleton and such measurements are of great use in forensic science, anatomy, and anthropometry. This study was an attempt to formulate a linear regression equation for estimation of stature by measuring the ulnar length in the living adult Khasi population.

Methods

The study population consists of 164 subjects (Male: 118; Female: 46) between 25 and 45 years of age. The left and right ulnar lengths were measured from the tip of the olecranon process to the tip of the styloid process with the elbow flexed and palm spread over the opposite shoulder by a spreading caliper. The measurements of the stature of the volunteers were done in the standard anatomical standing position with a bared foot with the head in the Frankfort plane. The documented data were calculated by the standard statistical software.

Results

The height and ulnar length in males (160.85 ± 6.34 cm and 24.41 ± 1.10 cm, respectively) were found to be significantly (p < 0.001) higher than females (149.56 ± 2.95 cm and 22.58 ± 0.47 cm, respectively). Significant positive correlation coefficient (r) between height (cm) and ulnar length (cm) were observed in both males (r = 0.955, P < 0.001) and females (r = 0.915, P < 0.001), respectively.

Conclusion

The length of the ulna provides an accurate and reliable means in estimating the height of an individual; being almost a percutaneous bone, its length can be measured easily. The regression formulae that were derived in this study will be useful for clinicians, human anatomists, archeologists, anthropologists, and forensic experts.

## Introduction

In a living being, growth is a dynamic vital process. Of course, physical growth becomes static after reaching the adult stage. In humans, physical growth is usually measured by measuring the height of an individual. The height of an individual can be achieved by toting up the length of certain bones that are directly involved in stature (i.e., the cranium, vertebral column, first sacral vertebra, femur, tibia, talus, and calcaneus) [1]. On the other hand, the height of an individual can be estimated from different parts of the body by doing an anthropometric study of the skeleton. This is an area of interest to human anatomists, anthropologists, and forensic experts [2].

Individual recognition is one of the most important components in the medico-legal field. A person can be identified by examining the different types of characteristics of the person. The age, sex, and height of an individual are three different characteristics that mostly reveal the identity of an individual. Among these, the height of a person is definitely one of the most important and useful aspects to focus on for the identity of a missing person [3]. Anthropometric characters have a direct association with the sex, shape, and appearance of an individual. These factors are closely related to each other and with the appearance of internal structure and tissue components, which are sequentially influenced by environmental and genetic factors [4]. In children, the measurement of height is one of the essential tools for the assessment of nutrition and growth as well as to predict pulmonary functions and to calculate the body surface area [5]. Moreover, the accurate measurement of body height along with bodyweight is also very essential to evaluate the nutritional status and pharmacokinetic parameters [6-7]. But in certain situations, varieties of physical disabilities may jeopardize the process of accurate measurement of height in a standing position [8].

Since ancient times, different scientists have put forward different equations across the globe to estimate the height of different groups of populations by using different parts of the skeleton. The length of the arm span is one of the most acceptable parameters to assess height [5]. Allbrook D et al., in their analysis, put forward standards for the estimation of stature by using ulnar length among the British and East American male population [9]. In the same way, Krogman WM and Iscan MY gave the most details about the estimation of stature from skeletal remains [10].

It has been observed that the formulae and equations put forward by different workers for the estimation of height by using the different anthropometric parameters are exclusively population and region-specific. This study wanted to determine whether the measurement of ulnar length could be reliably and reproducibly measured among the adult Khasi population of Meghalaya, India, and to develop prediction equations for height from the anthropometric measurement.

## Materials and methods

Study design

The present study was an observational prospective study carried out on 164 (Male: 118; Female: 46) healthy Khasi staff members in the 25-45 years age group working in North Eastern Indira Gandhi Regional Institute of Health and Medical Sciences (NEIGRIHMS), Shillong, between January 2014 to December 2014. The abovementioned age group was selected as the ossification of all bones is completed by the age of 25 years, the height of an individual decreases due to the aging process after the age of 45 years, and most women experience postmenopausal osteoporosis after the age of 45 years. Subjects with definite physical disabilities that might affect their height and/or bone length (e.g. those with a spinal deformity or past history of spinal surgery, in a wheelchair, or stooping or walking with a limp) were excluded.

Anthropometric measurements

Anthropometric measurements were taken from the above-mentioned normal healthy volunteers by a team of trained faculty members and resident doctors of the department of anatomy, NEIGRIHMS. The height was taken in the standing position following standard anthropometric protocols [11]. The height of the volunteers was measured in the standard standing anatomical position with bare feet and keeping the head in the Frankfort horizontal plane. Height was measured from crown to heel by using a stadiometer. The ulnar length was measured in the same position percutaneously with the help of a widespread caliper from the tip of the olecranon process to the tip of the ulnar styloid process with the elbow flexed and palm spread over the opposite shoulder. One of the important components of anthropometric measurements is precision and reliability, thus each measurement was taken by the same researcher twice and the mean of them was taken as the true value for the corresponding observation. To avoid any diurnal variation, the measurements were taken at the morning session from 8 AM to 11 AM [12].

Statistical analyses

The collected data of ulnar length and respective height were analyzed statistically by using Microsoft Office Excel 2007 and Microsoft Graph Chart software (Microsoft Corporation, Redmond, WA). The mean and standard deviations (SD) of the data were calculated for both anthropometric variables. A comparison of the mean of body height and ulnar length between the sexes was carried out using a student t-test. The relationship between body height and ulnar length was determined by the Pearson correlation coefficient. Linear regression analysis was done to examine the extent to which ulnar length can reliably predict body height. Finally, these relationships were plotted as a scatter diagram. Statistical significance was set at p-value < 0.05.

Ethical approval was obtained from the institutional board vide letter No. NEIGR/IEC/2013/45, and informed written consent was taken from all participants in the study.

## Results

During the study period from January 2014 to December 2014, a total of 164 healthy personnel were included. Of these participants, 118 (72%) were male and 46 (28%) were female. The age of the participants ranged from 25 to 45 years.

The height and ulnar length in males (160.85 ± 6.34 cm and 24.41 ± 1.10 cm, respectively) were found to be significantly (p < 0.001) higher than in females (149.56 ± 2.95 cm and 22.58 ± 0.47 cm, respectively) (Table 1).

**Table 1:** Anthropometric parameters in males and females expressed as mean ± standard deviation

Parameters	Male (n=118)	Female (n=46)	Significance
Height (cm)	160.85±6.34	149.56±2.95	P<0.001*
Ulnar length (cm)	24.41±1.10	22.58±0.47	P<0.001*
Mean of height and ulnar length ratio ± standard deviation	6.59±0.089	6.62±0.055	

Descriptive statistics of height and ulnar length (cm) in both males and females are shown in Table 1. Statistically significant (p < 0.001) differences were found in the height and ulnar length between males and females. Mean height and ulnar length did not show significant variation among the different genders (p > 0.05).

On correlation and regression analyses between body height and ulnar length among the participants, it was observed that the Pearson correlation coefficient (r) between height (cm) and ulnar length (cm) had a significant positive correlation in both males (r = 0.955, P < 0.001) and females (r = 0.915, P < 0.001) participants i.e. if the length of the ulna increases or decreases, the height of the participants also increases or decreases (Table 2).

**Table 2:** Correlation and regression analysis between body height and ulnar length among male and female study subjects

Parameters	Male	Female
Correlation Coefficient (r)	0.955 (P<0.001*)	0.915 (P<0.001*)
Regression Equation	Height=5.495 x Ulnar length + 26.71, R^2^ = 0.913	Height=5.777 x Ulnar length + 19.09, R^2^ = 0.836

Pearson correlation coefficients (r) between body height and ulnar length among the participants are shown in Table 2. All the correlation coefficients were found to be statistically significant (p < 0.001). Thus the length of the ulna is positively and significantly related to the height of the participants.

The regression equation found in this study was as follows:

For adult Khasi male: Height=5.495 x Ulnar length + 26.71; R2 = 0.913

For adult Khasi female: Height=5.777 x Ulnar length + 19.09; R2 = 0.836

*Correlation coefficient significant at 0.01% level of significance

**Figure 1:**
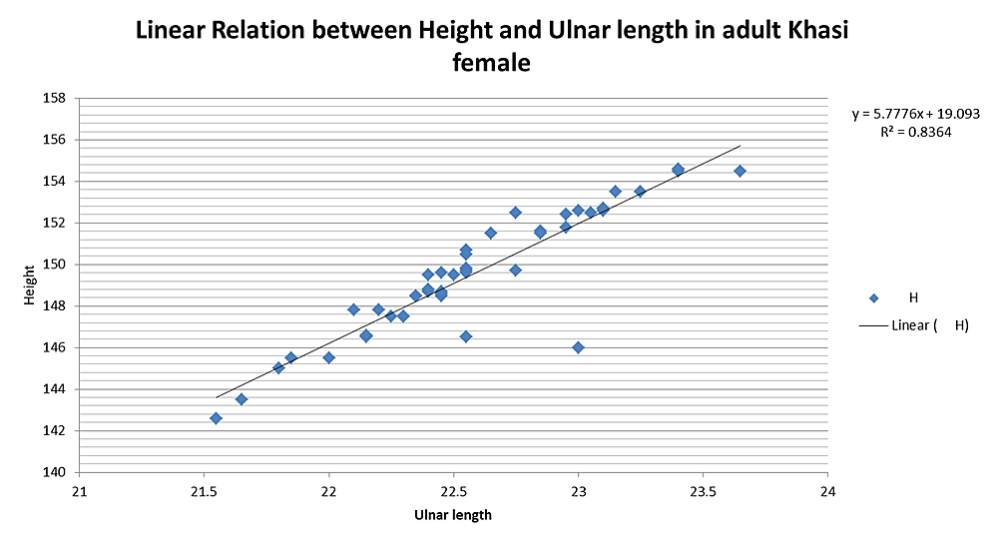
Linear relation between height and ulnar length in adult Khasi females

**Figure 2:**
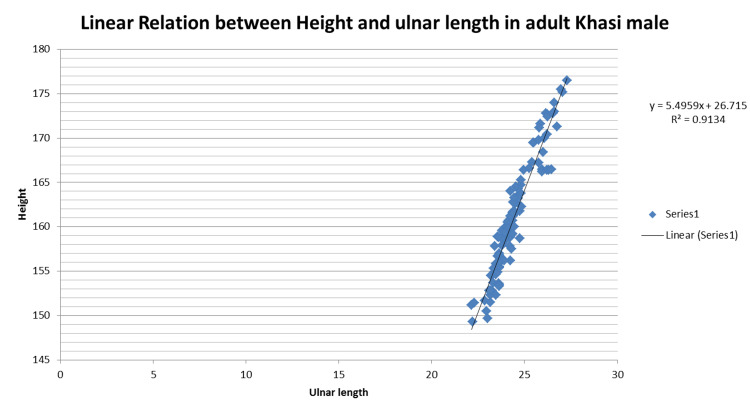
Linear relation between height and ulnar length in adult Khasi males

On further analysis of the linear regression plot of height and ulnar length in female and male study subjects, (Figures 1-2), it has been observed that the regression formula within a region also varies between the male and female populations.

## Discussion

Across the globe as well as in India, many researchers used the arm span and long bones of individuals of different age groups to estimate the height of an individual [1-5,8-9,13-29]. The ulnar length is considerably easier and better than arm span [19] and length of hand [20] for the calculation of height. In an earlier study, researchers observed that racial variation was very evident in the correlation of length of ulna and standing height in Asian, White, and Black populations [21]. Several studies from India have reported that the length of the ulna of individuals of different age groups can be used as a proxy tool to determine the height of the individuals [2,18,22-24,26-28].

It is observed that no scientific research work has been carried out to study the relationship between the ulnar length and standing height among the ethnic people of Meghalaya. In our observation, we found that the length of the ulna could be used as a proxy apparatus for the assessment of the standing height of a person (p˂0.001). In our study, it was observed that the length of the ulna was less than the corresponding standing height in both genders. The result of our study is in agreement with contemporary research reported by Anupriya A et al. [22], Kumar et al. [23] from India, and Gui H et al. [24] from Pakistan.

The results of our research work show that there is noteworthy affirmative involvement among the length of the ulna and erect height in men (r = 0.955, P < 0.001) and women (r = 0.915, P < 0.001), which are reasonably similar to the available established literature. The ulna length-height relationship has also been established by Barbosa VM et al., in his recent analysis found in the Portuguese population a value of r = 0.748 in men and r = 0.703 in women [17]. Similar findings regarding the relationship between ulnar length and erect posture have also been observed by Purushotham CN et al. (with a value of r = 0.689 in men and r = 0.99 in women) [23] and Borhani-Haghighi M. et al. (with a value of r = 0.59 in men and r = 0.57 in women) [29].

The use of the regression equation has immense importance in those individuals where erect height cannot be calculated in a direct way for limb deformities, amputated legs or shortening of lower limbs, various vertebral column deformities, or in bedridden patients. In these situations, we can use the ulnar length in the regression equation for the measurement of the height of an individual. The measurements of height are vital for calculating the basic calorie requirements, physical capacity, dosage adjustments for drugs, as well as for the identification of an individual.

Madden AM et al. opined that the ulnar length can be a proxy tool for the measurement of height in the different ethnic groups of people [21]. In this context, Cook et al. viewed that the estimation of height by using regression equations will not be 100% accurate [30]. The estimation of height from the ulnar length is comparatively easier over the use of other anthropometric parameters particularly in bedridden patients where proper positioning of the lower limb may be difficult [30].

## Conclusions

In conclusion, we revealed that the physical measurement of the length of the ulna significantly correlated with the height of the study participants. Thus, these measurements may be reliable predictors for the stature estimation of the adult Khasi population of Meghalaya, India.
